# Increased EMR2 expression on neutrophils correlates with disease severity and predicts overall mortality in cirrhotic patients

**DOI:** 10.1038/srep38250

**Published:** 2016-12-01

**Authors:** Chien-Hao Huang, Wen-Juei Jeng, Yu-Pin Ho, Wei- Teng, Yi-Chung Hsieh, Wei-Ting Chen, Yi-Cheng Chen, Hsi-Hsien Lin, I-Shyan Sheen, Chun-Yen Lin

**Affiliations:** 1 Department of Gastroenterology and Hepatology, Chang-Gung Memorial Hospital, Linkou Medical Center, Taiwan; 2 Graduate Institute of Clinical Medical Sciences, College of Medicine, Chang-Gung University, Taoyuan, Taiwan; 3 School of Medicine, College of Medicine, Chang-Gung University, Taoyuan 333, Taiwan; 4 Department of Microbiology and Immunology, College of Medicine, Chang Gung University, Taoyuan 333, Taiwan; 5 Chang Guang Immunology Consortium, Chang Gung Memorial Hospital and Chang Gung University, Taoyuan 333, Taiwan; 6 Department of Anatomic Pathology, Chang Gung Memorial Hospital- Linkou, Taoyuan 333, Taiwan

## Abstract

Patients with liver cirrhosis are susceptible to infections with high short-term mortalities. One CD97-related EGF-TM7 molecule, EMR2 (EGF-like molecule containing mucin-like hormone receptor 2), had been shown to regulate human neutrophil function, potentiate systemic inflammation. Nevertheless, EMR2 could also suppress neutrophil survival. Studying the role of EMR2 on neutrophil would be intriguing. 48 healthy volunteers and 100 cirrhotic patients were enrolled. Neutrophils were isolated from peripheral blood and cell surface markers were measured by flow cytometry.EMR2 expression levels correlated with CTP scores and increased further in patients with infections. These EMR2-expressed neutrophils were with activated phenotype, but with deranged functions like increased resting oxidative burst and impaired phagocytosis ability. Ligation of EMR2 could increase the phagoburst capacity but not the phagocytosis ability. Furthermore, neutrophils with higher EMR2 expression were more apoptotic and lost the LPS-induced neutrophil survival. Finally, EMR2 expressions on neutrophils correlated with infections and their levels greater than 25 had an AUC = 0.708 for predicting mortality. In conclusion, EMR2 expression levels correlated with CTP scores and increased further in cirrhotic patients with infections. These high EMR2-expressed neutrophils had activated phenotype but with deranged functions. Higher levels of these EMR2-expressed neutrophils correlated with infectious complications and predict mortality.

Cirrhosis represents a late stage of progressive hepatic fibrosis characterized by distortion of the hepatic architecture and the formation of regenerative nodules^1^. Patients with cirrhosis are susceptible to a variety of complications, and their life expectancy can be markedly reduced, especially in those with acute on chronic liver failures^2^,^3^. Infections, especially bacterial infections, account for the major causes for decompensating liver cirrhosis^4^ or acute on chronic liver failure, and contribute towards high morbidity and mortality of these patients^5^,^6^.

Several studies stress that immune paralysis, reflected as elevated serum levels of both pro-inflammatory and anti-inflammatory cytokines, is the major immune dysfunction in patients with severe cirrhosis and related to the subsequent bacterial infections^7^. Therefore, early recognition of high-risk patients and appropriate management accordingly, especially to those who are awaiting liver transplantation or suffering from spontaneous bacterial peritonitis (SBP), could improve survival^8^,^9^.

In recent years, evidence for the role of CD97-related EGF-TM7 molecule, EMR2 (EGF-like molecule containing mucin-like hormone receptor 2) in the migration and adhesion of myeloid cell during cell differentiation, maturation and activation has emerged^10^. The ligation of EMR2 could increase neutrophil adhesion, migration and anti-microbial mediator production and could enhance the systemic inflammation^11^. Furthermore, other studies had shown that EMR2 expression on the neutrophil increased in patients with SIRS and correlated with the extent of organ failure^12^. Its gene variant could sensitize mast cells to IgE-independent vibration-induced degranulation^13^. On the contrary, evidences for EMR2 expression on neutrophils associated with sepsis^14^ and ligation of EMR2 could suppresses LPS-induced neutrophil survival had been revealed^15^. From this point of view, these EMR2 expressed neutrophils could play a dual role in inflammation and possibly are responsible for the immune dysregulation, a major pathogenic event in sepsis^16^. Similar to the neutrophil behavior in sepsis, an interesting observation also demonstrated a paradoxical behavior of neutrophils with both high resting phagoburst activity and poor phagocytosis ability from cirrhotic patients^17^,^18^,^19^. Therefore, the role of EMR2 expression on the neutrophils in the cirrhotic patients is intriguing. But studies on the relationship between EMR2, neutrophil and its relationship to the severity of cirrhotic patients are lacking^20^.

The purpose of this study was to evaluate the role of EMR2 on the neutrophil in patients with cirrhosis and to correlate their expression to the outcome of these cirrhotic patients.

## Materials and Methods

### Patients

This study protocol conformed to the ethical guidelines of the 1975 Declaration of Helsinki and was approved by the ethical committees of Chang Gung Memorial Hospital. All the enrolled participants had given the signed informed consent.

Normal controls (NC) without any apparent diseases or infections were recruited from health check-up center in Chang Gung Memorial Hospital.

Patients with LC were recruited from outpatient clinic or liver wards of the Chang Gung Memorial Hospital if they met inclusion and exclusion criteria. Patients who were categorized as having LC were enrolled into study if they met the following criteria: (i) LC based on a histopathological diagnosis or compatible laboratory data, clinical features, and sonographic/computed tomography/magnetic resonance imaging findings; (ii) no evidence for obvious hepatocellular carcinoma or other metastatic liver tumor; and (iii) no immunosuppressive medication including oral corticosteroids within the last 6 months prior to study entry. None of the patients received blood products within at least 7 days prior to study inclusion. Blood samples for analysis of standard biochemical parameters and immunological parameters were drawn during admission or during regular visits in the outpatient clinic. Exclusion criteria includes: (i) age younger than 18; (ii) acute alcoholic hepatitis or acute on chronic liver failure without previous documented LC history; (iii) etiology for cirrhosis was unknown or other than hepatitis B virus, hepatitis C virus, and alcohol; (iv) refusal to participate in the study; (v) concurrent evidence of sepsis; (vi) uncontrolled diabetes mellitus (HbA1c > 7%).

Totally, 48 healthy volunteers (34 male, 14 female; Mean age: 51 ± 10 years) and 100 cirrhotic patients (77 male, 23 female; Mean age: 55 ± 13 years, 26 patients in Child-Turcotte-Pugh (CTP) A group, 26 patients in CTP B group, and 48 patients in CTP C group) were enrolled in this study between 2008 and 2013 in our hospital. These patients were followed at least 1 year after enrolling or until death for their outcome analysis. Seven patients (one lost follow-up, six lost followed up less than a year) were excluded in the outcome analysis. Patients were managed with the up-to-date AASLD, APASL and EASL guidelines for liver cirrhosis and it’s complications. The antibiotics choices also conformed to these guidelines and our hospital’s Antimicrobial Susceptibility Testing.

### Study Design

After peripheral venous blood drawn from healthy volunteers and cirrhotic patients and collected with pyrogen-free tubes (BD Vacutainer lithium hepatin, BD, Plymouth, UK), they were sent for various laboratory examinations including blood cell counts, coagulation parameters, biochemistry, neutrophil function assays and level of EMR2 expression on neutrophils immediately. Child-Turcotte-Pugh (CTP) classification and score were calculated. The occurrence of bacterial infectious complications, including spontaneous bacterial peritonitis, urinary tract infections, pneumonia, etc., which require admissions or ER visits, and the mortalities of these study subjects were recorded.

### Blood sampling

Neutrophils drawn from the peripheral blood of our subjects were isolated by PolymorphPrep^**TM**^ (Axis–Shield PoC, Oslo, Norway) and washed three times with freshly phosphate- buffered saline (PBS) (Sigma Aldrich, St. Louis, MO) at 4 °C and suspended in RPMI-1640 (Sigma Lifesciences, USA).

### Flow cytometry

The neutrophils were stained with fluorescein 5-isothiocyanate (FITC)-labeled anti-human CD11b, CD49d, CD181, and CD182 from BD Biosciences for 30 min in 70 μl of PBS at room temperature. After staining, cells were washed with 5 ml of the flow cytometry buffer and resuspended in the flow cytometry buffer supplemented with 1% paraformaldehyde (Electron Microscopy Sciences). Background staining as negative control was assessed after optical average zero standardized. Data were acquired and analyzed using BD FACSCalibur, CellQuest (Becton Dickinson, San Jose, CA, USA) and FlowJo software (Treestar Inc., Palo Alto, CA). The calculations were first estimated using PRISM version 5.00 (GraphPad Software, Inc., San Diego, CA, USA) then confirmed by SPSS.

### Effect of EMR2 on Neutrophil Phagocytosis and Oxidative Burst

We also evaluate the metabolic activity of oxidative burst *in vitro* and the neutrophil ability to phagocytize E. coli *in vitro*. It is a common method in the estimation of neutrophil phagocytosis by the determination of the percentage of phagocytizing cells and the ability to phagocytize opsonized bacteria. The assessment of phagocytosis was performed using the Phagotest Kit (ORPEGEN Pharma, Germany) containing fluorescein-labeled opsonized Escherichia coli (E. coli - FITC), according to the manufacturer’s instructions. The oxidative burst ability of neutrophil was evaluated by Oxidative burst kit (Orpegen Pharma, Heidelberg, Germany) and analyzed by flow cytometry by the fluorescence intensity of R123, according to the manufacturer’s instructions.

### Neutrophil phagoburst and phagocytosis activities determined by negative control, incubation with E. coli, ligation of EMR2 using EMR2 antibody, and both E-coiI and ligation of EMR2

All the isolated neutrophils from healthy volunteers and cirrhotic patients were incubated with or without EMR2 antibody. The function of ligating neutrophil with EMR2 antibody in a cirrhotic patient was supposed to increase the adhesion, production of reactive oxygen species which is likely to cause bystander damage, promote endothelial dysfunction and multi-organ failure. After mixing the isolated neutrophils with or without anti-EMR2 antibody (2A1), the Phagoburst and Phagotest (ORPEGEN Pharma, Germany) were tested.

### Statistical analysis

Statistical analysis was performed using the GraphPad Prism software (GraphPad Prism 5; Inc., USA) and SPSS software (SPSS17.0; SPSS Inc., Chicago, IL, USA).

Continuous variables were expressed as means ± SD, while categorical variables were expressed as frequencies or percentages. Groups were compared using independent t-tests for continuous variables between two groups and one-way anova for that between four groups. Multiple variable analyses were performed using either multiple linear or logistic regression analysis. Correlations were determined using Pearson’s correlation test. The Kaplan-Meier method and log-rank test were used for survival analysis.

## Results

### Increased expression of EMR2 on neutrophils correlated with the severity of liver cirrhosis

48 healthy volunteers (34 male, 14 female; mean age:51 ± 10 years old) and 100 cirrhotic patients (69 male, 23 female; mean age: 55 ± 13 years old: 26 patients in CTP A group, 26 patients in CTP B group, and 48 patients in CTP C group) were enrolled in this study. There were no differences among these groups in terms of gender, age, and etiologies (Table 1). The white blood cell counts and absolute neutrophil count did not decrease significantly in these groups, too (Table 1).

**Figure 1:**
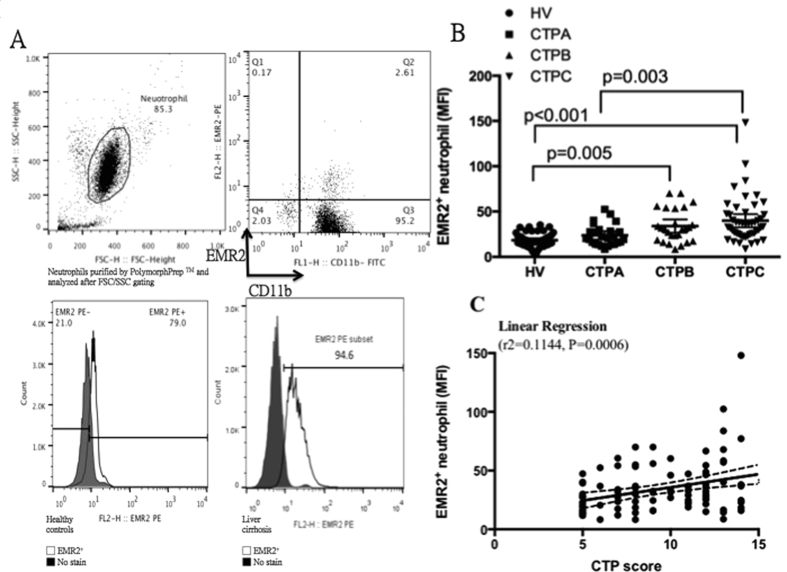
The levels of EMR2 expressions on neutrophil correlated with different cirrhotic stage (**A**) Neutrophils were purified by PolymorphPrep^**TM**^ (Axis–Shield PoC, Oslo, Norway) and washed three times with freshly phosphate- buffered saline (PBS) (Sigma Aldrich, St. Louis, MO) at 4 °C and suspended in RPMI-1640 (Sigma Lifesciences, USA). By neutrophil-specific FSC/SSC gating strategy and reconfirmed by neutrophil phenotypic marker CD 11b (>97% cell purity, Fig. 1A upper part), measurements of the EMR2 expressions on neutrophils from healthy controls and liver cirrhotic patients were done as compared to the negative control (Fig. 1A lower part). The mean MFI of this isotype control was less than 10. That means when the MFI above 10, it would be positive for the EMR-2 expression. (**B**) When sub-grouping cirrhotic patents according to CTP class, an increasing EMR2 expression on neutrophil was noted as the severity of liver cirrhosis progress when comparing that with healthy volunteers (MFI of EMR2: Child-Turcotte-Pugh A vs. B vs. C vs. healthy volunteers: 23.74 ± 11.15 vs. 33.97 ± 18.00 vs. 39.89 ± 24.95 vs. 18.38 ± 7.74; p < 0.0001) (**C**) The expression level of EMR2 on neutrophil significantly correlated with CTP scores by linear regression (r2 = 0.1144, P = 0.0006)

Neutrophils were purified by PolymorphPrep TM, gated by neutrophil-specific FSC/SSC strategy, and reconfirmed by neutrophil phenotypic marker CD 11b (>97% cell purity, Fig. 1A upper part). Measurements of the EMR2 expressions on neutrophils from healthy controls and liver cirrhotic patients were done as compared to the negative control by the flow cytometric analysis (Fig. 1A lower part). The mean MFI of this isotype control was less than 10. That means when the MFI above 10, it would be positive for the EMR-2 expression. The neutrophils from patients with liver cirrhosis had significantly higher EMR2 expressions than that from healthy volunteer (MFI of EMR2: 34.15 ± 21.35 vs. 18.38 ± 7.74, *P* < 0.0001). When sub-grouping these cirrhotic patents according to Child-Turcotte-Pugh classification, an increasing EMR2 expression on neutrophil was noted as the severity of liver cirrhosis progress when comparing that with healthy volunteers (MFI of EMR2: Child-Turcotte-Pugh A vs. B vs. C vs. healthy volunteers: 23.74 ± 11.15 vs. 33.97 ± 18.00 vs. 39.89 ± 24.95 vs. 18.38 ± 7.74; p < 0.0001, Fig. 1B and Table 1). The expression level of EMR2 on neutrophil significantly correlated with CTP scores by linear regression (r2 = 0.1144, P = 0.0006, Fig. 1C).

### Cirrhotic patients with infectious complications has significant higher EMR2 expression on neutrophils than their counterparts

In addition, previous studies have shown that bacterial complications in cirrhotic patients correlated with the severity of cirrhosis and the dysfunctions of neutrophils^17^,^21^.

Firstly, the type of infectious complications these patients suffered from was analyzed. Totally 41 of the 100 subjects suffered from infectious complications that required ER visits or admissions. Most common infections were as following: ① 13/41 patients infected with pneumonia (culture: E. coli > MSSA > MRSA = Citrobactei freundi stenotrophomonus maltophilia) ② 12/41 patients infected with spontaneous bacterial infections (culture: E. coli > K. penumonia > Enterobacter. Cloacae) ③ 10/41 patients infected with urinary tract infections (culture: E. coli ≫  Enterococcus faecalis) ④ 5/41 bacteremia (culture: E. coli > salmonella = MSSA = Enterococcus faecalis) ⑤ 1/41 patients infected with cellulitis.

**Figure 2:**
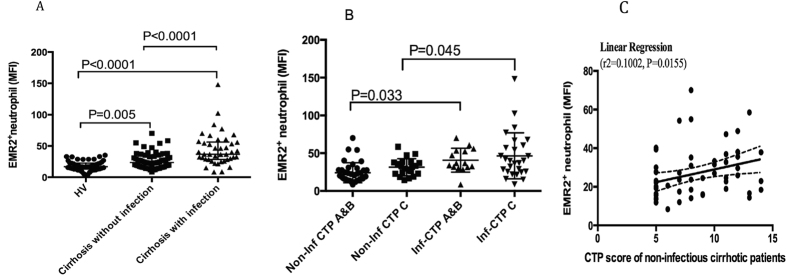
EMR2 expressions on neutrophils increased significantly in cirrhotic patient, especially in those with infectious complications (**A**) EMR2 expression on neutrophils increased significantly in cirrhotic patients with infectious complications than cirrhotic patients without infectious complications (MFI of EMR2: 44.40 ± 26.05 vs. 26.73 ± 13.03, p < 0.001); Cirrhotic patients with or without infections both had significantly higher EMR expression on their neutrophils than that of healthy volunteers (MFI of EMR2 on HVs: 18.38 ± 7.74, p < 0.05) (**B**) Both CTP A&B and CTP C cirrhotic patients with infectious complications had significantly higher EMR2 expression on neutrophils than their counterparts. (**C**) The expression levels of EMR2 on neutrophils in cirrhotic patients without infectious complications also correlated with CTP scores significantly by linear regression (r2 = 0.1002, P = 0.015).

Secondly, we analyzed the relationship between EMR2 expression on neutrophil and the bacterial complications. As shown in Fig. 2A, EMR2 expression on neutrophils increased significantly in cirrhotic patients with infectious complications than cirrhotic patients without infectious complications (MFI of EMR2: cirrhotic patients with infections vs. cirrhotic patients without infection: 44.40 ± 26.05 vs. 26.73 ± 13.03, p < 0.001). Cirrhotic patients with or without infections both had significantly higher EMR expression on their neutrophils than that of healthy volunteers (MFI of EMR2 on HVs: 18.38 ± 7.74, p < 0.05). When further subgrouping cirrhotic patients with or without infections into different CTP classifications as showed in Fig. 2B, both CTP A&B and CTP C cirrhotic patients with infectious complications had significantly higher EMR2 expression on neutrophils than their counterparts.

It is noteworthy that the expression levels of EMR2 on neutrophils in cirrhotic patients without infectious complications also correlated with CTP scores significantly by linear regression (r2 = 0.1002, P = 0.015, Fig. 2C).

### High EMR2-expressed neutrophils from cirrhotic patients were activated and with increased resting oxidative burst but impaired phagocytosis ability

**Figure 3:**
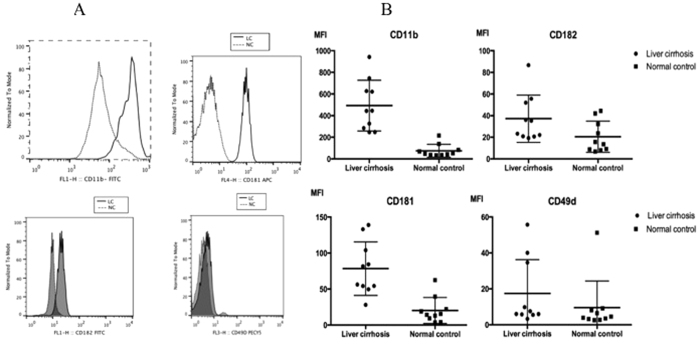
EMR2 expressed neutrophils were with activated phenotype reflected as high CD11b, CD181, CD182 and CD49d expression. (**A**) Comparison by histogram (**B**) Comparison by MFI. The EMR2-expressed neutrophils from liver cirrhotic patients did express higher levels of the activation molecules, including CD11b, CD181, CD182 and CD49d, than those from normal controls.

The next issue that we would like to address was the phenotypes of these EMR2-expressed neutrophils. As shown in (Fig. 3A,B), the EMR2-expressed neutrophils from liver cirrhotic patients did express higher levels of the activation molecules, including CD11b, CD181, CD182 and CD49d, than those from normal controls.

**Figure 4:**
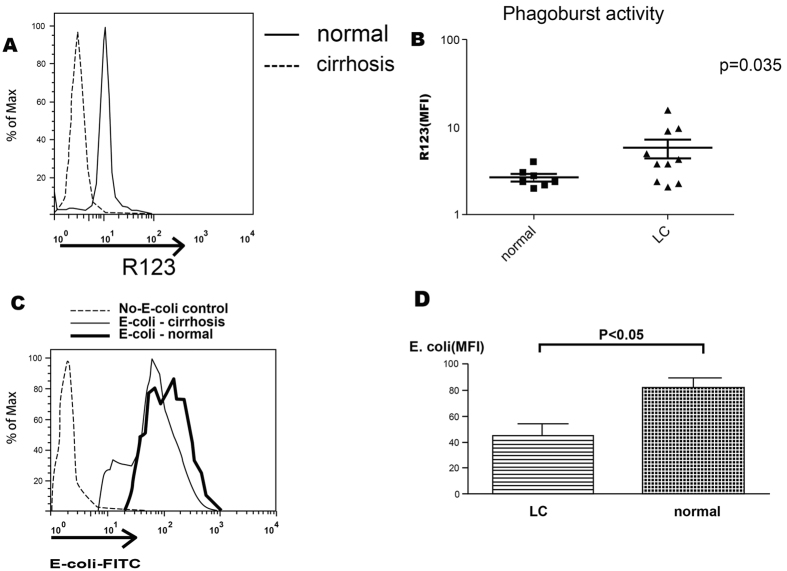
High EMR2-expressed neutrophils from cirrhotic patients were activated and with increased resting oxidative burst but impaired phagocytosis ability. (**A**,**B**) Significantly higher resting oxidative burst capacity of neutrophils from cirrhotic patients than that from healthy volunteers (p = 0.035). (**C**,**D**) The phagocytosis ability of neutrophils was impaired in cirrhotic patients when compared to that with healthy volunteers (p < 0.05).

We then investigated the functional aspects of these neutrophils. We harvested the neutrophils from Child C cirrhotic patients due to their highest EMR2 expression levels and healthy volunteers respectively. Similar to previous reports^17^, there were significantly higher resting oxidative burst capacity of neutrophils from cirrhotic patients than that from healthy volunteers (p = 0.035, Fig. 4A,B). However, the phagocytosis ability of neutrophils was impaired in cirrhotic patients when compared to that with healthy volunteers (Fig. 4C,D).

### Ligation of EMR2 could not rescue the impaired phagocytosis ability of neutrophils from cirrhotic patients

**Figure 5:**
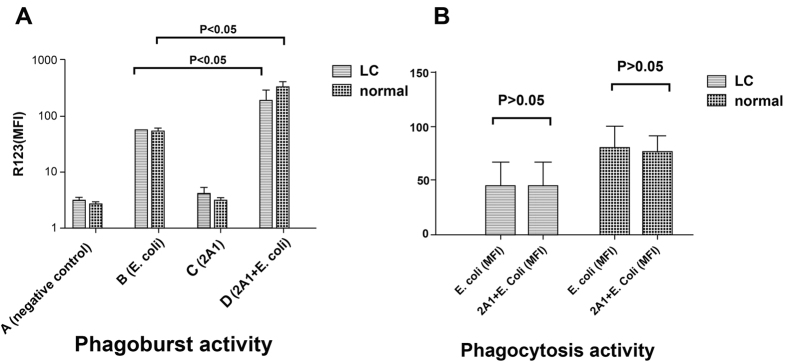
Ligation of EMR2 could not rescue the impaired phagocytosis ability of neutrophils from cirrhotic patients. (**A**) EMR2 ligation (2A1) could increase the phagoburst capacity in both normal volunteers and cirrhotic patients under E-coli incubation (**B**) EMR2 ligation (2A1) could not rescue phagocytosis ability in both normal volunteers and cirrhotic patients.

Previous study supported the role of EMR2 in potentiating neutrophil activation and further inflammatory response. Using an anti-EMR2 antibody, ligation of EMR2 increases neutrophil adhesion and migration, and augments superoxide production and proteolytic enzyme degranulation^22^. We had showed the impaired phagocytosis abilities of neutrophils in cirrhotic patients. By ligation of EMR2 with an anti-EMR2 mAb, we tried to improve the phagocytosis ability. However, as shown in Fig. 5A, EMR2 ligation could increase the phagoburst capacities in both normal volunteers and cirrhotic patients with E-coli incubation, but not in the impaired phagocytosis abilities in normal volunteers and cirrhotic patients (Fig. 5B).

### High EMR2-expressed neutrophils from cirrhotic patients represented an apoptotic phenotype and could not be rescued by opsonized E. coli incubation

Neutrophils play a key role in the elimination of pathogens. They are remarkably short-lived with a circulating half-life of 6–8 hours^23^. Their survival and correlation with EMR2 expression without/with opsonized E. coli incubation was studied.

**Figure 6:**
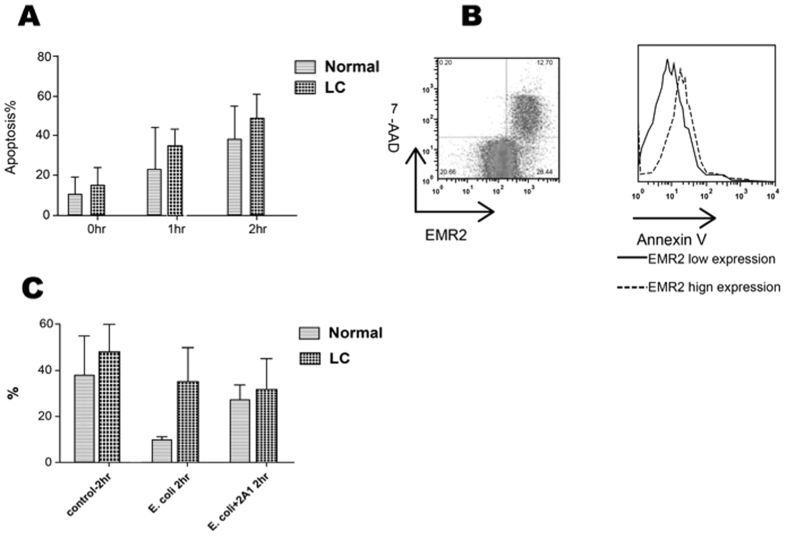
High EMR2-expressed neutrophils from cirrhotic patients represented an apoptotic phenotype and could not be rescued by opsonized E. coli incubation. (**A**) Neutrophils from cirrhotic patients were more apoptotic than from healthy volunteers. (**B**) Neutrophils with high expression of EMR2 were also with high expression of annexin-V and high percentages of 7-AAD (**C**) Neutrophil from healthy volunteers or cirrhotic patients were incubated respectively with opsonized E. coli without/with anti-EMR2 antibody (2A1). E-coli could prolong the survival of neutrophils from healthy volunteers (decreased % of apoptosis) but not from cirrhotic patients. Ligation of EMR2 reversed the effect of neutrophil survival prolongation by E-coli from healthy volunteers but not from cirrhotic patients.

Neutrophil from both healthy volunteers and cirrhotic patients were cultured in RPMI for 1 and 2 hours. The apoptotic percentage was evaluated by Annexin-V expression at 0 hr, 1 hr, and 2 hr respectively using flow cytometry. As shown by Fig. 6A, neutrophils from cirrhotic patients were more apoptotic than from healthy volunteers. Neutrophils with high expression of EMR2 were also with high expression of annexin-V and high percentages of 7-AAD (Fig. 6B). Neutrophil from healthy volunteers or cirrhotic patients (Fig. 6C) were incubated respectively with opsonized E. coli without/with anti-EMR2 antibody (2A1). The apoptotic percentages were measured by annexin-V at 2 hours of incubation. The data showed that E-coli could prolong the survival of neutrophils from healthy volunteers but not from cirrhotic patients. Ligation of EMR2 in healthy volunteers but not in cirrhotic patients reversed the effect of neutrophil survival prolongation with E-coli incubation.

### Levels of EMR2 expression on neutrophils correlate with infectious complications and could predict the mortality of cirrhotic patients

**Figure 7:**
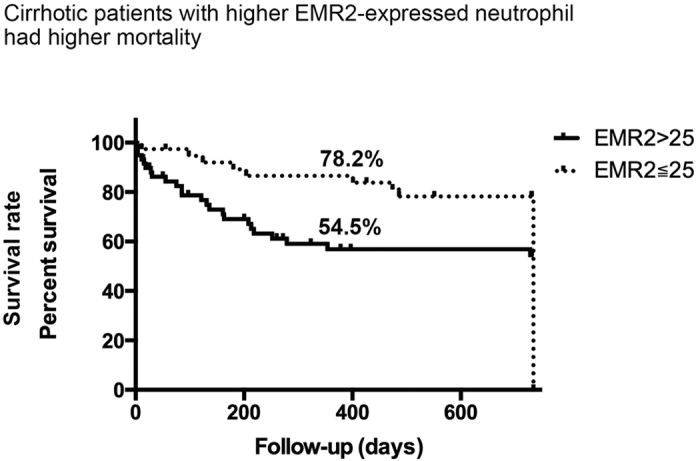
Levels of EMR2 expression on neutrophils correlate with infectious complications and could predict the mortality of cirrhotic patients. Kaplan-Meier survival curve showed significant difference between patients with EMR2 expression levels on neutrophils greater than 25(MFI) and less or equals to 25(MFI) (54.5% vs. 78.2%, p = 0.0136). The EMR2 expression levels on neutrophils greater than 25 had a sensitivity of 86.8% and a specificity of 54.8% for predicting mortality (AUC = 0.708, P value = 0.000).

To further clarify the impact of the EMR2 expression on the disease outcome, we had prospectively followed these cirrhotic patients for two years. The mean follow-up days were 534.47 ± 30.48 days. At the end of follow-up, the mortality rate was 66%. There was a linear relationship between the EMR2 expression levels on neutrophils and mortality (linear regression, r^2^ = 0.130, P = 0.000). As revealed by Fig. 7, the Kaplan-Meier survival curve showed significant difference between patients with EMR2 expression levels on neutrophils greater than 25(MFI) and less or equals to 25(MFI) (54.5% vs. 78.2%, p = 0.0136). The EMR2 expression levels on neutrophils greater than 25 had a sensitivity of 86.8% and a specificity of 54.8% for predicting mortality (AUC = 0.708, P value = 0.000). In addition, we also found a positive correlation between EMR2 expression level and infectious complications (r = 0.423, p = 0.000). As for EMR2’s prediction of infectious complications, it also showed significance (OR: 1.058, p = 0.000, 95% CI: 1.027–1.090), in addition to CTP and MELD scores.

## Discussion

Our results highlighted the role of EMR2 expression on neutrophils of liver cirrhotic patients. EMR2 expression levels correlated with CTP scores and increased further in cirrhotic patients with infections. These EMR2-expressed neutrophils were with activated phenotype as reflected by higher level of the activation molecules CD11b, CD181, CD182 and CD49d, but with deranged functions such as increased resting oxidative burst and impaired phagocytosis ability. The ligation of EMR2 could increase the phagoburst capacity but not the phagocytosis ability in cirrhotic patients. Furthermore, neutrophils with higher EMR2 expression were more apoptotic and lost the LPS-induced neutrophil survival. Finally, we showed that the higher levels of these EMR2-expressed neutrophils could predict the mortality of these cirrhotic patients.

Cirrhosis-associated immune dysfunction (CAIDs) refers to the main syndromic abnormalities of immune function, immunodeficiency and systemic inflammation that are present in cirrhosis^24^. When cirrhosis progressed from compensated to decompensated cirrhosis or ACLF, the CAID phenotype switched from a pro-inflammatory to an immunodeficient phenotype. Previous EMR2 studies found that EMR2 ligation can potentiate neutrophils at the “early stage” of inflammation, including changes in cell adhesion molecules and cell morphology, enhanced chemotaxis, production of anti-microbial mediators and degranulation^22^. Leemans *et al*. had reported *in vivo* ligation of CD97 in a murine model of colitis, which increased mortality due increased bacterial infection^25^. However, Lewis SM *et al*. recently demonstrated that EMR2 is a potential biomarker for SIRS and it is associated with the extent of organ failure^26^. Child C or decompensated end-stage liver disease often leads to multiple organ failure^4^,^27^. Although oxidative burst (production of reactive oxygen) of neutrophils is a major host defense mechanism to kill invading microorganisms, its excessive activities had been shown to be associated with SIRS and the extent of organ failure^18^,^28^. These findings are consistent with our observation of high resting oxidative burst activities in Child C cirrhotic patients, which could be further enhanced by EMR2 ligations. In addition, these EMR2-expressed neutrophils expressed higher level of the activation molecules CD11b, CD181, CD182 and CD49d, and correlated with infectious complications, which partly corresponded to previous studies that the expressions of CD11b and CD49d elevated in neonatal infections or sepsis respectively^29^,^30^. The most common infectious complications including pneumonia, SBP and UTI also echoed previous works^31^,^32^.

It has been demonstrated that chronic endotoxemia correlate with the severity of liver disease^33^ and contribute to immune paralysis in liver cirrhotic patients^7^,^34^. Several studies stress that immune paralysis is fundamental for sepsis in patients with severe cirrhosis^7^. Our study found neutrophils were more apoptotic in cirrhotic patients than in healthy volunteers and their phagocytosis abilities were impaired. Neutrophils with higher EMR2 expressions in these cirrhotic patients were even more apoptotic implies that EMR2 is also involved in the “late stage” of neutrophil activation, namely the resolution phase and related to more rapid resolution of inflammation and immune dysfunction^20^. E. coli could prolong neutrophil survival in healthy volunteers but not in those cirrhotic patients with E-coli plus EMR2 ligation, suggesting the “exhausted” neutrophils by chronic endotoxemia in cirrhotic patients indeed play a role in anti-inflammation and immune dysfunction. Recently Sagiv JY had *et al*.^35^ had documented phenotypic diversity and plasticity in circulating neutrophil subpopulations in lung and breast cancers. Our findings are in accord with their observation that neutrophils in cirrhosis could play opposite roles concurrently.

Cirrhotic patients with higher EMR2-expressed neutrophils had higher mortalities were also demonstrated in the study. The EMR2 expression levels on neutrophils greater than 25 had a sensitivity of 86.8% and a specificity of 54.8% for predicting mortality (AUC = 0.708, P value = 0.000). With an AUC > 0.7, it is confirmed the prediction ability of this parameter^36^. However, we did not intend to compare the prediction ability of different parameters. What we intended to show is the relationship between the expression level of EMR2 and mortality prediction in order to highlight the importance of these EMR2-expressed neutrophils in cirrhotic patients due to their deranged functions such as increased resting oxidative burst and impaired phagocytosis ability as we had demonstrated in the study. These might increase mortalities in liver cirrhosis patients.

Several studies had also showed the importance of neutrophil dysfunction on survival in liver cirrhosis: Neutrophil phagocytosis capacity (NPC) predicted survival in stable cirrhosis and their resting OB (oxidative burst) also predicted 90-day mortality18; Impaired neutrophil phagocytic activity in the acute liver failure and subacute liver failure cohorts on admission predicted non-survival without liver transplantation^37^. Our findings that these higher EMR2-expressed neutrophils in cirrhotic patients had increased resting oxidative burst but decreased phagocytosis abilities, correlation with infectious complications and higher mortalities provides another important parameter to predict the outcome of cirrhotic patients.

## Conclusion

EMR2 expression levels correlated with CTP scores and increased further in cirrhotic patients with infections. These high EMR2-expressed neutrophils were with activated phenotype, but with deranged functions. By EMR2 ligation under E-coli incubation, it could increase the phagoburst capacity, but not increase the phagocytosis ability. Furthermore, neutrophils with higher EMR2 expression were more apoptotic and their survival could not be rescued by E. coli co-culture. Finally, higher levels of these EMR2-expressed neutrophils correlated with infectious complications and could predict mortality of these cirrhotic patients.

## Additional Information

**How to cite this article**: Huang, C.-H. *et al*. Increased EMR2 expression on neutrophils correlates with disease severity and predicts overall mortality in cirrhotic patients. *Sci. Rep.* **6**, 38250; doi: 10.1038/srep38250 (2016).

**Publisher’s note:** Springer Nature remains neutral with regard to jurisdictional claims in published maps and institutional affiliations.

## Figures and Tables

**Table 1:** Demographic characteristics of healthy volunteers and patients with liver cirrhosis.

Parameters	Healthy Volunteers (n = 48)	Liver cirrhosis			
		Child A (n = 26)	Child B (n = 26)	Child C (n = 48)	P value
Male, n (%)	34 (70.8)	20 (76.9)	20 (76.9)	37 (77.1)	0.883
Age, M ± SD (years)	51.6 ± 10.7	55.8 ± 9.9	57.2 ± 14.9	53.5 ± 13.8	0.263
Etiology					0.656
Alcohol		10 (38.5)	10 (38.5)	17 (35.4)	
HBV		12 (46.2)	8 (30.8)	21 (43.8)	
HCV		4 (15.4)	8 (30.8)	10 (20.8)	
Child-Turcotte-Pugh score		5.1 ± 0.3	7.9 ± .0.7	12.2 ± 1.3	**<0.001**
MELD score		8.8 ± 4.7	12.3 ± 5.8	22.6 ± 10.7	**<0.001**
Bilirubin total (mg/dL)	0.90 ± 0.33	1.18 ± 0.42	2.39 ± 3.55	10.36 ± 10.58	**<0.001**
Albumin (g/dL)	4.69 ± 0.27	4.0 ± 0.6	3.1 ± 0.5	2.7 ± 0.4	**<0.001**
WBC* (×100/*μ*l)	61.16 ± 14.14	59.94 ± 30.09	50.06 ± 26.58	66.38 ± 33.10	0.413
ANC* (×100/*μ*l)	35.47 ± 10.83	38.22 ± 21.42	35.75 ± 21.68	47.47 ± 26.60	0.307
Platelets (×1000/*μ*l)	230.06 ± 59.38	163.05 ± 97.15	84.18 ± 59.87	87.07 ± 66.16	**<0.001**
AST (U/L)	23.23 ± 9.92	64.25 ± 57.35	60.43 ± 51.97	92.00 ± 65.34	**<0.001**
ALT (U/L)	25.93 ± 12.51	47.62 ± 45.04	30.60 ± 17.55	48.96 ± 45.43	**0.003**
INR	1.03 ± 0.08	1.12 ± 0.11	1.38 ± 0.21	1.86 ± 0.79	**<0.001**
Cr (mg/dL)	0.94 ± 0.22	1.24 ± 1.25	1.31 ± 1.26	1.53 ± 1.31	**0.011**
EMR2 Neutrophil (MFI)	18.38 ± 7.74	23.74 ± 11.55	33.97 ± 18.00	39.89 ± 24.95	**<0.001**

48 healthy volunteers (34 male, 14 female; mean age: 51 ± 10 years old) and 100 cirrhotic patients (69 male, 23 female; mean age: 55 ± 13 years old: 26 patients in CTP A group, 26 patients in CTP B group, and 48 patients in CTP C group) were enrolled in this case-control cohort study. There were no differences among these groups in terms of gender, age, and etiologies. The white blood cell counts (WBC) and absolute neutrophil count (ANC) did not decrease significantly in these groups, too.

^*^WBC, ANC: subjects without infections counted.
